# Analysis of Prognostic Factors Affecting the Brain Metastases Free Survival and Survival After Brain Metastases in Breast Cancer

**DOI:** 10.3389/fonc.2020.00431

**Published:** 2020-04-03

**Authors:** Weikai Xiao, Xuerui Li, Anli Yang, Bo Chen, Shaoquan Zheng, Guochun Zhang, Wenju Deng, Ning Liao

**Affiliations:** ^1^Department of Breast Cancer, Cancer Center, Guangdong Provincial People's Hospital, Guangdong Academy of Medical Sciences, Guangzhou, China; ^2^Department of Breast Oncology, Sun Yat-sen University Cancer Center, State Key Laboratory of Oncology in South China, Collaborative Innovation Center for Cancer Medicine, Guangzhou, China

**Keywords:** breast cancer brain metastases, BMFS, SABM, receptor change, prognosis

## Abstract

This study aimed to analyze the factors affecting brain metastases free survival (BMFS) and the survival after brain metastases (SABM). The data of 215 patients with breast cancer brain metastases (BCBM) in Sun Yat-sen University Cancer Center from January 2000 to August 2017 were retrospectively analyzed. The clinicopathological features of BCBM were analyzed, and their effects on BMFS and SABM were analyzed by univariate and multivariate COX regression. Finally, it was analyzed whether the receptor status of the brain metastases and the primary lesions were consistent. The median age of the entire cohort was 46 years old. The median BMFS, SABM and overall survival were 31, 9 and 44.2 months, respectively. Clinical stage, molecular subtypes and bone metastasis were independent prognostic factors affecting BMFS. TNM stage IV (HR, 4.99 [95% CI, 2.13–11.7]) and triple negative subtype (HR, 2.06 [95% CI, 1.35–3.14]) was significantly associated with shorter BMFS, but the presence of bone metastases (HR, 0.63 [95% CI, 0.45–0.88]) was a favorable factor for BMFS. Molecular subtypes, resection of BCBM and whole brain radiotherapy (WBRT) were independent factors for SABM. The triple negative subtype (HR, 2.02[95% CI, 1.12–3.64]) was significantly associated with shorter SABM. However, resection of BCBM (HR, 0.31 [95% CI, 0.15–0.65]) and WBRT (HR, 0.57 [95% CI, 0.35–0.93]) were independent factors in improving SABM. The conversion rate of ER was 11.1%, PR was 29.6%, and HER2 was 3.7% between paired breast cancer and brain metastases. BMFS and SABM have different influencing factors. Resection of BCBM and WBRT can significantly improve SABM. The frequency of HER2 status changes between the paired BCBM and the primary lesions is low.

## Introduction

Despite significant advances in the diagnosis and treatment of breast cancer, metastasis is still an important factor that seriously affects patients' quality of life and prognosis. It has been reported that distant metastases have been found at the initial diagnosis of breast cancer in about 6–10% of patients (1). In addition, 30 to 40% of patients with early-stage breast cancer will have recurrence and metastasis during the postoperative follow-up period and progress to advanced breast cancer (2). The bones, lungs, liver and brain are the four most common distant metastatic sites of breast cancer (3). Compared with bone metastases and visceral metastases, patients with brain metastases have significantly worse prognosis (4).

It is worth noting that the incidence of brain metastases seems to be increasing in recent years. The possible reason is that with the advancement of medical treatment (such as new chemotherapy drugs, targeted treatment, etc.), extracranial diseases can be better controlled. However, macromolecular drugs cannot enter the brain because of the blood-brain barrier, which greatly increases the chance of brain metastases. In addition, the progress of brain imaging technology has also increased the detection rate of brain metastases. At present, the incidence of brain metastases from breast cancer ranks second, accounting for about 10 to 16%, second only to that of lung cancer (5, 6).

Multi-disciplinary therapy (MDT) is the first choice for the treatment of brain metastases in breast cancer (5, 7). MDT for breast cancer brain metastasis includes surgery, whole brain radiation therapy (WBRT), stereotactic radiosurgery (SRS), chemotherapy, endocrine therapy, targeted therapy, etc. (5). For patients with multiple brain metastases and neurological symptoms, WBRT combined with palliative care is the preferred option (5, 8). In general, patients with brain metastases of 3 or less were recommended to receive surgical resection. Patients with 4 to 5 brain metastases but <3 cm in diameter can undergo SRS (9). Previous studies have shown that chemotherapy combined with WBRT can further increase the survival time of patients (10). For brain metastases in HER2-positive breast cancer, trastuzumab combined with sequential anti-HER2 targeted drugs (such as lapatinib, TDM-1) can significantly improve survival (11).

The main purpose of this study was to analyze which clinicopathological factors affect the occurrence of brain metastases and which factors affect the survival after the occurrence of brain metastases. This may help identify patients at high risk for brain metastases and patients with poor prognosis after brain metastases, thus providing them with some preventive or therapeutic measures. Finally, it is a controversial issue whether the receptor status of brain metastases is consistent with that of the primary tumor. Therefore, we analyzed the consistency of the receptor status between brain metastases and primary tumors.

## Materials and Methods

The main clinical and pathological variables collected include: age at first diagnosis, histological type, clinical stage, histological grade, ER, PR, and HER2 status, time of brain metastasis, and survival time after brain metastasis. Clinical staging was performed according to the 7th edition of the TNM staging of breast cancer promulgated by the American Joint Committee on Cancer (AJCC). ER, PR, and HER2 status was assessed by immunohistochemistry or *in situ* hybridization analysis as previously described (12). A positive ER or PR status is defined as ≥1% of tumor cells with immunostaining. HER2 overexpression/amplification was determined as a 3+ immunohistochemical score (>30% homogeneous and intense membrane staining of tumor cells) or a positive *in situ* hybridization result. As previously reported, results for ER, PR, and HER2 status were used as surrogate markers for the classification of major breast cancer subtypes, including the luminal (HER2^−^) type (ER^+^ and/or PR^+^), HER2-positive, and triple-negative (ER^−^/PR^−^/HER2^−^) subtype.

We only included women with breast cancer diagnosed pathologically. The exclusion criteria were as follows: (1) male breast cancer; (2) patients with incomplete clinical and pathological data; (3) patients without follow-up; (4) patients with only carcinoma *in situ*.

## Diagnostic Criteria for Brain Metastases

1. BCBM confirmed by histopathology examination
2. When patients were unable to obtain a pathological examination, we mainly diagnosed BCBM based on clinical manifestations and imaging examination. Clinical symptoms include one or more of the following: intracranial hypertension (headache, vomiting, and optic papillary edema), seizures, focal nerve dysfunction, cranial nerve invasion, and meningeal irritation. For patients with no clinical symptoms, the diagnosis of BCBM was confirmed by a breast oncologist and an imaging physician based on CT and MRI examinations.

## Patient Follow-Up Strategy

All breast cancer patients in our center were mainly followed up by telephone or outpatient clinic, and the follow-up results were recorded. The follow-up time started from the first diagnosis of breast cancer. Brain metastasis free survival (BMFS) was defined as the time from the first diagnosis of breast cancer to the discovery of brain metastases. The time after brain metastasis (SABM) was defined as the time from the diagnosis of brain metastasis to death or the last follow-up. Overall survival (OS) was defined as the time from the initial diagnosis of breast cancer to the death of the patient or the last follow-up.

## Statistical Methods

Statistical analysis was performed using SPSS 21.0 software package. Kaplan-Meier method was used for survival analysis, and log-rank test was used for comparison between groups. Multivariate analysis affecting survival was studied by Cox proportional hazard regression model. *P* < 0.05 indicates that the difference was statistically significant.

## Results

### Clinicopathological Features of BCBM Patients

Table 1 summarized the demographic data and tumor pathological characteristics of the 215 BCBM patients included in the study. 65.6% of patients were diagnosed with breast cancer between the ages of 40–59 years, with a median age of 46 years (21–73 years). The median follow-up time before brain metastasis was 31 months, and the median follow-up time after brain metastases was 9 months. Three people were lost to follow-up. The TNM stage of most patients was stage III (33.0%). 89.3% of patients were invasive ductal carcinoma, and the histological grade was mainly grade II and III. 34.9% of patients were of luminal (HER2^−^) type, 40.9% of patients were HER2 positive, 23.7% of patients were triple negative, and the remaining subtypes were unknown (0.5%). Brain metastasis was the first distant metastasis in 27.9% of patients. 32.1% of BCBM patients had bone metastases and visceral metastases. 28.4% of BCBM patients had visceral metastases but no bone metastases, and only 11.6% of BCBM patients had bone metastases but no visceral metastases. The median BMFS, SABM, and OS were 31, 9 and 44.2 months, respectively.

**Table 1:** Basic clinicopathological characteristics of BCBM patients.

**Clinicopathological features**	**No. (%)**
**Age at initial diagnosis**	
<40	57 (26.5)
40–59	141 (65.6)
≥60	17 (7.9)
**TNM stage**	
I	8 (3.7)
II	55 (25.6)
III	71 (33.0)
IV	42 (19.5)
Unknown	39 (18.1)
**Pathology type**	
Ductal	192 (89.3)
Lobular	6 (2.8)
Other	7 (3.3)
Unknown	10 (4.7)
**Histological grade**	
I	0 (0)
II	49 (22.8)
III	47 (21.9)
Unknown	119 (55.3)
**Molecular subtypes**	
Luminal (HER-)	75 (34.9)
HER2+	88 (40.9)
Triple negative	51 (23.7)
Unknown	1 (0.5)
**Bone metastasis**	
No	121 (56.3)
Yes	93 (43.3)
Unknown	1 (0.5)
**Liver metastases**	
No	135 (62.8)
Yes	78 (36.3)
Unknown	2 (0.9)
**Lung metastasis**	
No	108 (50.2)
Yes	107 (49.8)
**Number of brain metastasis**	
Single	41 (19.1)
Multiple	148 (68.8)
Unknown	26 (12.1)
**Surgical resection of brain tumor**	
Yes	42 (20)
No	172 (80)
**Whole brain radiotherapy**	
Yes	122 (56.7)
No	93 (43.3)
**Stereotactic surgery**	
Yes	54 (74.9)
No	161 (25.1)

### Influencing Factors of Brain Metastasis Free Survival (BMFS)

**Figure 1:**
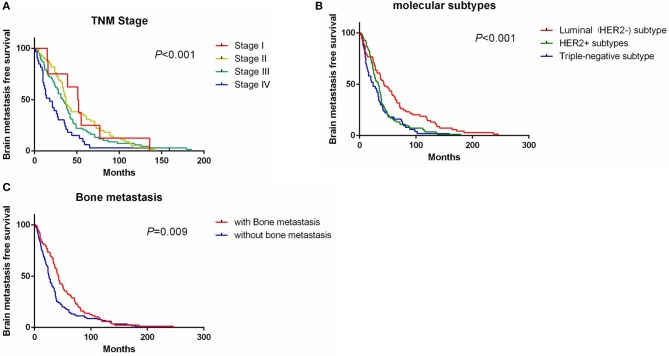
Survival curve of BMFS grouped by TNM stage **(A)**, molecular subtypes **(B)**, and bone metastasis status **(C)**. BMFS, brain metastases free survival.

Univariate analysis showed that clinical stage, molecular subtypes, bone metastases, and lung metastases were significantly associated with BMFS (Table 2). Patients with TNM stage IV had a shorter BMFS (HR, 4.99 [95% CI, 2.13–11.7], Figure 1A). For patients with stage II or III, their BMFS were not statistically different from that of stage I. Univariate analysis showed shorter BMFS for HER2-positive and triple-negative breast cancers, but multivariate analysis showed that only triple-negative subtypes were independent risk factors for BMFS (HR, 2.06 [95% CI, 1.35–3.14], Figure 1B). In addition, univariate analysis suggested that patients with bone metastases and lung metastases had longer BMFS, but after multivariate analysis, only patients with bone metastases showed significantly longer BMFS than patients without bone metastases (HR, 0.63 [95% CI, 0.45–0.88], Figure 1C).

**Table 2:** Univariate and multivariate analysis of Brain-metastasis free survival.

**Features**	**Univariate analysis**		**Multivariate analysis**	
	**HR (95%CI)**	***P***	**HR (95%CI)**	***P***
**Age**				
<40	1 (Reference)			
40–59	1.24 (0.90–1.71)	0.185		
≥60	1.72 (0.95–3.10)	0.074		
**TNM stage**				
I	1 (Reference)		1 (Reference)	
II	1.15 (0.55–2.42)	0.714	1.61 (0.73–3.59)	0.242
III	1.37 (0.66–2.86)	0.398	1.65 (0.75–3.66)	0.217
IV	2.35 (1.08–5.11)	0.031	4.99 (2.13–11.7)	<0.001
**Molecular subtypes**				
Luminal (HER-)	1 (Reference)		1 (Reference)	
HER2+	1.63 (1.17–2.26)	0.004	1.21 (0.83–1.75)	0.316
Triple negative	1.94 (1.34–2.83)	0.001	2.06 (1.35–3.14)	0.001
**Pathology type**				
Ductal	1 (Reference)			
Lobular	1.66 (0.68–4.07)	0.267		
Other	1.91 (0.89–4.08)	0.096		
**Histological grade**				
II	1 (Reference)			
III	0.82 (0.54–1.24)	0.346		
**Bone metastasis**				
No	1 (Reference)		1 (Reference)	
Yes	0.69 (0.52–0.12)	0.010	0.63 (0.45–0.88)	0.007
**Liver metastais**				
No	1 (Reference)			
Yes	0.86 (0.65–1.15)	0.313		
**Lung metastasis**				
No	1 (Reference)		1 (Reference)	
Yes	0.72 (0.55–0.95)	0.021	0.75 (0.53–1.05)	0.091

### Factors Affecting Survival After Brain Metastasis (SABM)

**Figure 2:**
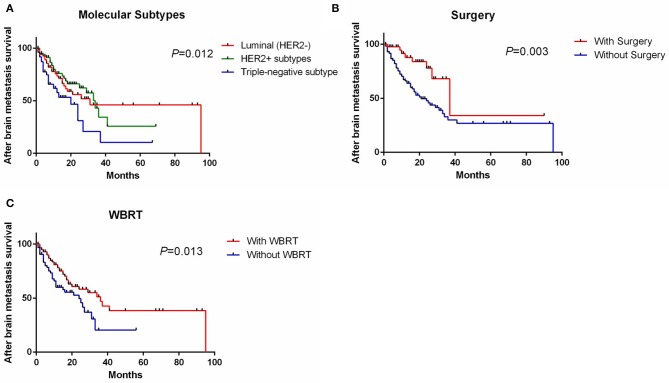
Survival curve of SABM grouped by molecular subtypes **(A)**, whether to undergo surgery **(B)**, and WBRT **(C)**. SABM, survival after brain metastases; WBRT, whole brain radiotherapy.

Univariate analysis showed that molecular subtypes, whether to undergo surgery and whole brain radiotherapy (WBRT) were significant prognostic factors affecting SABM (Table 3). Multivariate analysis showed that SABM in triple-negative patients (HR, 2.02 [95% CI, 1.12–3.64], Figure 2A) was significantly shorter than that in luminal type, but there was no statistical difference in SABM between HER2-positive and luminal (HER2^−^) patients. In addition, univariate and multivariate analysis suggested that surgical resection (HR, 0.31 [95% CI, 0.15–0.65], Figure 2B) and WBRT (HR, 0.57 [95% CI, 0.35–0.93], Figure 2C) were independent factors of SABM.

**Table 3:** Univariate and multivariate analysis of factors affecting survival after brain metastasis (SABM).

**Features**	**Univariate analysis**		**Multivariate analysis**	
	**HR (95%CI)**	***P***	**HR (95%CI)**	***P***
**Age**				
<40	1 (Reference)			
40–59	0.86 (0.51–1.45)	0.578		
≥60	1.29 (0.54–3.04)	0.568		
**TNM stage**				
I	1 (Reference)			
II	2.01 (0.27–15.1)	0.500		
III	4.31 (0.59–31.7)	0.151		
IV	4.34 (0.58–32.5)	0.153		
**Molecular subtypes**				
Luminal (HER2-)	1 (Reference)		1 (Reference)	
HER2+	0.92 (0.53–1.60)	0.756	0.89 (0.50–1.56)	0.671
Triple negative	2.04 (1.14–3.66)	0.017	2.02 (1.12–3.64)	0.012
**Pathology type**				
Ductal	1 (Reference)			
lobular	1.21 (0.38–3.87)	0.151		
other	1.61 (0.81–3.67)	0.422		
**Histological grade**				
II	1 (Reference)			
III	1.70 (0.74–3.91)	0.209		
**Bone metastasis**				
No	1 (Reference)			
Yes	0.65 (0.40–1.06)	0.081		
**Liver metastais**				
No	1 (Reference)		1 (Reference)	
Yes	1.65 (1.04–2.63)	0.035	1.34 (0.83–2.16)	0.228
**Lung metastasis**				
No	1 (Reference)			
Yes	0.94 (0.59–1.50)	0.800		
**Number of brain metastasis**				
Single	1 (Reference)			
Multiple	1.66 (0.97–2.85)	0.066		
**Surgical resection of brain tumor**				
No	1 (Reference)		1 (Reference)	
Yes	0.17 (0.04–0.70)	0.014	0.31 (0.15–0.65)	0.002
**Whole brain radiotherapy**				
No	1 (Reference)		1 (Reference)	
Yes	0.55 (0.5–0.89)	0.013	0.57 (0.35–0.93)	0.023
**Stereotactic surgery**				
No	1 (Reference)			
Yes	0.68 (0.40–1.14)	0.143		

### Changes of ER, PR and HER2 Status in Primary Breast Cancer and Brain Metastases

There were 3 patients had ER status change (1 positive to negative, 2 negatives to positive), 8 patients had PR status change (5 positives to negative, 3 negatives to positive), and 1 patient had HER2 status change (positive to negative) (Table 4).

**Table 4:** Changes of ER, PR, and HER2 status in primary lesions and brain metastases of breast cancer.

	**Pos–neg (%)**	**Neg–pos (%)**	**Total (%)**
ER (*n* = 27)	1 (3.7)	2 (7.4)	3 (11.1)
PR (*n* = 27)	5 (18.5)	3 (11.1)	8 (29.6)
HER2 (*n* = 27)	1 (3.7)	0 (0)	1 (3.7)

*Pos, positive; Neg, negative*.

### Discussion

The incidence of BCBM has been increasing in recent years and has become a major limitation on the survival and quality of life for many breast cancer patients. This study found that molecular subtypes significantly affect the occurrence and prognosis of brain metastases. Patients with triple negative subtypes had significantly shorter BMFS and SABM than luminal (HER2^−^) subtypes. However, there was no statistical difference in the effects of HER2-positive subtypes on BMFS and SABM compared with the luminal (HER2^−^) subtype. In addition, multivariate analysis showed that resection of brain metastases and WBRT can improve the prognosis of some patients with brain metastases. Finally, we also found that HER2 status conversions of primary and metastatic lesions rarely occur.

The clinical stage and molecular subtype of breast cancer were considered to be important factors affecting BCBM. In this study, it was not surprising to observe that patients with late clinical stage (IV) had a shorter BMFS because these patients had a large tumor burden and responded poorly to systemic therapy, which was also confirmed by previous studies (13). HER2-positive and triple-negative breast cancers are highly invasive breast cancers that are prone to metastasis, including visceral metastases and nervous system metastases (14). Anti-HER2 targeted therapy has greatly improved the natural course of HER2-positive breast cancer (15, 16). The prognosis of these patients after anti-HER2 treatment was similar to that of lumen-like breast cancer (14, 17). This study found that although BMFS in HER2-positive and triple-negative breast cancers was significantly shorter than the luminal type in univariate analysis, further multivariate analysis found that only triple-negative subtypes were independent prognostic factors affecting BMFS. This is consistent with the results reported by Sperduto PW et al. (18), and they also found that Basal and HER2 tumor subtypes have shorter BMFS. In addition, this study found that after the occurrence of brain metastasis, the triple negative subtype progressed rapidly and was an independent risk factor affecting SABM, but the effect of HER2-positive subtype on the prognosis was not statistically different from that of luminal subtype. Another study of Sperduto PW and colleagues also found that molecular subtypes were independent factors affecting SABM (19).

This study showed no significant difference in the effects of age on BMFS and SABM. Although young breast cancer is generally considered a high risk of recurrence and a poor prognosis (20), this effect was not observed in patients with brain metastases. In contrast, BMFS in patients older than 60 years appears to be shorter than breast cancer patients younger than 40 years, but there is no statistical difference. The possible reasons are that elderly patients are generally in poor condition, may have chronic diseases, and receive inadequate treatment. Purushotham A et al. found that patients older than 70 years of age with visceral metastasis had a higher risk of death than younger patients, with a median follow-up of 6.32 years for 3,553 patients (21). Visceral metastasis is considered to be a factor that affects the occurrence of brain metastases. We observed that patients with liver metastases had significantly shorter SABM than patients without liver metastases: HR: 1.65, 95% CI (1.04–2.63), but this effect was not found after multivariate analysis Statistical significance. In addition, we observed that patients with no bone metastases had significantly shorter BMFS than patients with bone metastases, which means that patients with bone metastases are often less prone to brain metastases. Patients with bone metastases are more hormone receptor positive, and hormone receptors are protective factors for the occurrence of brain metastases. The study by Li et al. (13) found that BMFS of lobular carcinoma was shorter than that of ductal carcinoma, but this trend was not observed in this study. Histological classification is also an important predictor of breast cancer prognosis. It was also considered to be an independent risk factor affecting SABM in the research by Li et al. However, this trend was not observed in this study, probably because of the histological classification of most patients was unknown in our cohort.

Brain metastases from breast cancer include local therapies (surgery, whole brain radiotherapy, and stereotactic radiosurgery) and systemic treatments (chemotherapy, endocrine therapy, and targeted therapy). Due to inconsistent chemotherapy regimens in most patients, while endocrine therapy was mainly used for hormone receptor-positive patients and targeted therapy was only used for HER2-positive patients. Therefore, in this study, the impact of local treatment on BCBM prognosis was mainly analyzed. WBRT was widely used in the treatment of brain metastases, but its ability to improve the prognosis was still controversial (26, 27). In this study, we observed whether or not receiving whole brain radiotherapy was an independent factor affecting SABM. The patients receiving whole brain radiotherapy had significantly better SABM than those who did not. In addition, SABM in the surgical group was significantly better than that in the non-surgical group. However, due to certain indications for surgery, patients undergoing surgery were generally in good general condition, single brain metastases, and no extracranial metastases. This may result in a better prognosis for this group of patients than other patients. Therefore, we performed a multivariable COX regression analysis and the selection bias was reduced to a certain extent. Surgical resection can improve prognosis if systemic disease is not present or is already controlled and KPS is 60 or higher, according to European Neuro-Oncology Association (EANO) guidelines (7). Stereotactic surgery was also a local treatment for brain metastases. It is less invasive than surgery and more accurate than whole brain radiotherapy (22). However, in this study, no improvement in the survival of patients with brain metastases was observed with stereotactic radiation therapy. Due to the unavailability of data, we did not assess the impact of Karnofsky Performance Status (KPS) on SABM, and previous studies have shown that KPS is an important factor affecting the prognosis of BCBM (23, 24).

In metastatic breast cancer, endocrine therapy and HER2-targeted treatment decisions were still largely based on the tissue characteristics of the primary tumor. However, some studies have found that the ER, PR, and HER2 status of metastatic lesions was inconsistent with that of the primary tumor. Therefore, we finally analyzed whether the receptor status between the matched primary and brain metastases was the same, and found that the inconsistency of PR was the most obvious, as high as 29.6%. The ER disagreement was 11.1%, while the HER2 disparity was the lowest at 3.7%. WAME et al. (25) conducted a large meta-analysis through 39 studies and found that the ER disparity between the paired brain metastases and the primary tumor was 20.8%, PR was 23.3%, and HER2 was 12.5%. The direction of receptor transformation is two-way. Possible reasons for the transition from positive to negative are treatment or tumor heterogeneity. Conversion from negative to positive may be due to problems with sampling during biopsy or clonal evolution during metastasis. Due to the existence of inconsistent receptors, some experts recommend biopsy of the metastases.

In conclusion, molecular subtypes significantly affect the occurrence and prognosis of brain metastases. The triple negative subtype means shorter BMFS and SABM. Brain metastasis surgery and WBRT might improve the prognosis of selected patients. The HER2 status of primary and metastatic lesions remained basically the same.

## Data Availability Statement

Data supporting the results of this study can be obtained from the corresponding author under reasonable request.

## Ethics Statement

The studies involving human participants were reviewed and approved by The Institutional Review Board of Guangdong Provincial People's Hospital. The patients/participants provided their written informed consent to participate in this study.

## Author Contributions

All authors participated in this research. WX and NL: concepts and design. WX, XL, SZ, and AY: data acquisition. WX, BC, and XL: data analysis and interpretation. WX, SZ, AY, GZ, and WD: material support. WX and NL: study supervision. WX, BC, and XL: writing, review, and revision of manuscripts. The final draft read and approved by all authors.

### Conflict of Interest

The authors declare that the research was conducted in the absence of any commercial or financial relationships that could be construed as a potential conflict of interest.

## References

[1] Siegel RL Miller KD Jemal A Cancer statistics, 2019. CA Cancer J Clin. (2019) 69:7–34. 10.3322/caac.21551 30620402

[2] Gonzalez-Angulo AM Morales-Vasquez F Hortobagyi GN. Overview of resistance to systemic therapy in patients with breast cancer. Adv Exp Med Biol. (2007) 608:1–22. 10.1007/978-0-387-74039-3_1 17993229

[3] Kennecke H Yerushalmi R Woods R Cheang MC Voduc D Speers CH. Metastatic behavior of breast cancer subtypes. J Clin Oncol. (2010) 28:3271–7. 10.1200/JCO.2009.25.9820 20498394

[4] Lin C Wu J Ding S Goh C Andriani L Lu S. Subdivision of M1 stage for *de novo* metastatic breast cancer to better predict prognosis and response to primary tumor surgery. J Natl Compr Canc Netw. (2019) 17:1521–8. 10.6004/jnccn.2019.7332 31805535

[5] Fontanella C De Carlo E Cinausero M Pelizzari G Venuti I Puglisi F. Central nervous system involvement in breast cancer patients: is the therapeutic landscape changing too slowly. Cancer Treat Rev. (2016) 46:80–8. 10.1016/j.ctrv.2016.03.014 27218867

[6] Costa R Carneiro BA Wainwright DA Santa-Maria CA Kumthekar P Chae YK. Developmental therapeutics for patients with breast cancer and central nervous system metastasis: current landscape and future perspectives. Ann Oncol. (2017) 28:44–56. 10.1093/annonc/mdw532 28177431 PMC7360139

[7] Soffietti R Abacioglu U Baumert B Combs SE Kinhult S Kros JM. Diagnosis and treatment of brain metastases from solid tumors: guidelines from the European Association of Neuro-Oncology (EANO). Neuro Oncol. (2017) 19:162–74. 10.1093/neuonc/now241 28391295 PMC5620494

[8] Tsao MN Xu W Wong RK Lloyd N Laperriere N Sahgal A. Whole brain radiotherapy for the treatment of newly diagnosed multiple brain metastases. Cochrane Database Syst Rev. (2018) 1:CD003869. 10.1002/14651858.CD003869.pub4 29365347 PMC6491334

[9] Yamamoto M Serizawa T Higuchi Y Sato Y Kawagishi J Yamanaka K. A multi-institutional prospective observational study of stereotactic radiosurgery for patients with multiple brain metastases (JLGK0901 study update): irradiation-related complications and long-term maintenance of mini-mental state examination scores. Int J Radiat Oncol Biol Phys. (2017) 99:31–40. 10.1016/j.ijrobp.2017.04.037 28816158

[10] Nieder C Marienhagen K Dalhaug A Aandahl G Haukland E Pawinski A. Impact of systemic treatment on survival after whole brain radiotherapy in patients with brain metastases. Med Oncol. (2014) 31:927. 10.1007/s12032-014-0927-2 24647787

[11] Krop IE Lin NU Blackwell K Guardino E Huober J Lu M. Trastuzumab emtansine (T-DM1) versus lapatinib plus capecitabine in patients with HER2-positive metastatic breast cancer and central nervous system metastases: a retrospective, exploratory analysis in EMILIA. Ann Oncol. (2015) 26:113–9. 10.1093/annonc/mdu486 25355722 PMC4679405

[12] Wen J Yang Y Liu P Ye F Tang H Huang X. Development and validation of a nomogram for predicting survival on the base of modified lymph node ratio in breast cancer patients. Breast. (2017) 33:14–22. 10.1016/j.breast.2017.01.017 28259045

[13] Li R Zhang K Siegal GP Wei S. Clinicopathological factors associated with survival in patients with breast cancer brain metastasis. Hum Pathol. (2017) 64:53–60. 10.1016/j.humpath.2017.03.022 28428107

[14] Xiao W Zheng S Yang A Zhang X Zou Y Tang H. Breast cancer subtypes and the risk of distant metastasis at initial diagnosis: a population-based study. Cancer Manag Res. (2018) 10:5329–38. 10.2147/CMAR.S176763 30464629 PMC6225920

[15] Goutsouliak K Veeraraghavan J Sethunath V De Angelis C Osborne CK Rimawi MF. Towards personalized treatment for early stage HER2-positive breast cancer. Nat Rev Clin Oncol. (2020) 17:233–50. 10.1038/s41571-019-0299-9 31836877 PMC8023395

[16] Moasser MM Krop IE. The Evolving Landscape of HER2 Targeting in Breast Cancer. JAMA Oncol. (2015) 1:1154–61. 10.1001/jamaoncol.2015.2286 26204261

[17] Martin AM Cagney DN Catalano PJ Warren LE Bellon JR Punglia RS. Brain metastases in newly diagnosed breast cancer: a population-based study. JAMA Oncol. (2017) 3:1069–77. 10.1001/jamaoncol.2017.0001 28301662 PMC5824221

[18] Sperduto PW Kased N Roberge D Chao ST Shanley R Luo X. The effect of tumor subtype on the time from primary diagnosis to development of brain metastases and survival in patients with breast cancer. J Neurooncol. (2013) 112:467–72. 10.1007/s11060-013-1083-9 23462853

[19] Sperduto PW Kased N Roberge D Xu Z Shanley R Luo X. Effect of tumor subtype on survival and the graded prognostic assessment for patients with breast cancer and brain metastases. Int J Radiat Oncol Biol Phys. (2012) 82:2111–7. 10.1016/j.ijrobp.2011.02.027 21497451 PMC3172400

[20] Partridge AH Hughes ME Warner ET Ottesen RA Wong YN Edge SB. Subtype-dependent relationship between young age at diagnosis and breast cancer survival. J Clin Oncol. (2016) 34:3308–14. 10.1200/JCO.2015.65.8013 27480155

[21] Purushotham A Shamil E Cariati M Agbaje O Muhidin A Gillett C. Age at diagnosis and distant metastasis in breast cancer–a surprising inverse relationship. Eur J Cancer. (2014) 50:1697–705. 10.1016/j.ejca.2014.04.002 24768572

[22] Shi S Sandhu N Jin MC Wang E Jaoude JA Schofield K Stereotactic radiosurgery for resected brain metastases: single-institutional experience of over 500 cavities. Int J Radiat Oncol Biol Phys. (2019) 105:E90 10.1016/j.ijrobp.2019.06.2266 31785338

[23] Sperduto PW Mesko S Li J Cagney D Aizer A Lin NU Beyond an Updated Graded Prognostic Assessment (Breast GPA): a prognostic index and trends in treatment and survival in breast cancer brain metastases from 1985 to today. Int J Radiat Oncol Biol Phys. (2020) 19:S0360-3016(20)30205-4. 10.1016/j.ijrobp.2020.01.051 PMC7276246 32084525

[24] Griguolo G Jacot W Kantelhardt E Dieci MV Bourgier C Thomssen C. External validation of modified breast graded prognostic assessment for breast cancer patients with brain metastases: a multicentric european experience. Breast. (2018) 37:36–41. 10.1016/j.breast.2017.10.006 29073498

[25] Schrijver W Suijkerbuijk K van Gils CH van der Wall E Moelans CB van Diest PJ. Receptor conversion in distant breast cancer metastases: a systematic review and meta-analysis. J Natl Cancer Inst. (2018) 110:568–80. 10.1093/jnci/djx273 29315431

[26] Pasquier D Darlix A Louvel G Fraisse J Jacot W Brain E. Treatment and outcomes in patients with central nervous system metastases from breast cancer in the real-life ESME MBC cohort. Eur J Cancer. (2019) 125:22–30. 10.1016/j.ejca.2019.11.001 31835235

[27] Brown PD Ahluwalia MS Khan OH Asher AL Wefel JS Gondi V Whole-brain radiotherapy for brain metastases: evolution or revolution. J Clin Oncol. (2018) 36:483–91. 10.1200/JCO.2017.75.9589 29272161 PMC6075843

